# What should a robot disclose about me? A study about privacy-appropriate behaviors for social robots

**DOI:** 10.3389/frobt.2023.1236733

**Published:** 2023-12-15

**Authors:** Manuel Dietrich, Matti Krüger, Thomas H. Weisswange

**Affiliations:** ^1^ Honda Research Institute Europe GmbH, Offenbach, Germany; ^2^ Honda Research Institute Japan Co Ltd., Saitama, Japan

**Keywords:** social robot, robot mediator, privacy, disclosure, vignette approach

## Abstract

For robots to become integrated into our daily environment, they must be designed to gain sufficient trust of both users and bystanders. This is in particular important for social robots including those that assume the role of a mediator, working towards positively shaping relationships and interactions between individuals. One crucial factor influencing trust is the appropriate handling of personal information. Previous research on privacy has focused on data collection, secure storage, and abstract third-party disclosure risks. However, robot mediators may face situations where the disclosure of private information about one person to another specific person appears necessary. It is not clear if, how, and to what extent robots should share private information between people. This study presents an online investigation into appropriate robotic disclosure strategies. Using a vignette design, participants were presented with written descriptions of situations where a social robot reveals personal information about its owner to support pro-social human-human interaction. Participants were asked to choose the most appropriate robot behaviors, which differed in the level of information disclosure. We aimed to explore the effects of disclosure context, such as the *relationship* to the other person and the *information content*. The findings indicate that both the information content and relationship configurations significantly influence the perception of appropriate behavior but are not the sole determinants of disclosure-adequacy perception. The results also suggest that expected benefits of disclosure and individual general privacy attitudes serve as additional influential factors. These insights can inform the design of future mediating robots, enabling them to make more privacy-appropriate decisions which could foster trust and acceptance.

## 1 Introduction

Current research in human-robot interaction (HRI) is increasingly focusing on scenarios where robots interact with multiple people (Sebo et al., 2020) as compared to the dyadic robot-human configuration which has been the predominant paradigm in the past. It is widely agreed that designing robots for an interaction with groups, teams, coworkers, or social communities requires new approaches which go beyond established means for classical HRI (Clabaugh and Matarić, 2019; Sebo et al., 2020). Group interaction scenarios are particularly relevant for the field of social robotics. Social robots are intended to support individuals not only as companions or personal assistants but also by proactively initiating or maintaining interpersonal relationships, or by encouraging individuals to participate in social activities (Chita-Tegmark and Scheutz, 2021; Coghlan, 2021; Erel et al., 2022b). Personalized robotic coaches and companions have shown positive psychological effects on individuals, either through social presence (Thunberg et al., 2020), reactive behavior (Wada et al., 2003), mental assistance (Cao et al., 2019), or by providing positive interventions fostering self-awareness and gratitude (Jeong et al., 2020).

In applications with multiple people, social robots are often characterized as a mediator, moderator, or facilitator (hereafter referred to as “robot mediators”) with the goal to positively shape interactions and relationships between people (Abrams and der Pütten, 2020; Weisswange et al., 2023). Robot mediators are characterised as entities that provide support for individuals living together or help to establish and sustain relationships with outsiders, such as family members or friends who do not live in the same household (Jeong et al., 2020). A robot can actively participate as an explicit group member (Chita-Tegmark and Scheutz, 2021), subtly influence interaction dynamics (Tickle-Degnen et al., 2014; Traeger et al., 2020) or offer explicit means to initiate (Erel et al., 2022a) or enhance a remote connection (Yang and Neustaedter, 2018; Tennent et al., 2019; Einecke and Weisswange, 2022). A number of robot mediators has also been designed for group discussions, where a robot moderated speaking times (Tennent et al., 2019) and conflict situations between younger children (Shen et al., 2018), or enhanced inter-personal emotional support (Erel et al., 2022b). The possibilities of robots to facilitate inter-generational interaction have been explored through a study with co-located preschool children and care home residents (Joshi and Šabanović, 2019).

In many applications, person-related information, such as activities and behaviors, emotional and cognitive states, or high-level information like user preferences, are or will be needed for effective mediation. A robot might have to be aware of schedules, goals and mutual interests to provide support in social constellations. In contrast to dyadic human-robot interaction, where similar data might be collected and used, this data could now be, explicitly or implicitly, disclosed to other users. However, to our knowledge, challenges for data protection and privacy expectations of participants have not been researched. This includes users’ expectations on how their personal information can be shared or transferred between people, what can be disclosed for the purpose of mediation, and how people can maintain control about this information flow.

The field of computer-supported collaborative work (CSCW) (Grudin and Poltrock, 1997) previously investigated technology-supported mediation or cooperation and also discussed privacy considerations (e.g., Chen and Xu, 2013; Wong and Mulligan, 2019). However, in CSCW, the mediation technology is predominantly conceptualized as a tool (Wallace et al., 2017; Sebo et al., 2020) and not as an embodied agent. What we know from the shared usage of mediation tools might not apply for robots as mediators. People who are confronted with a robot counterpart might have (privacy) expectations that are predominantly led by prior experience with what is expected from humans in similar roles. However, what we know from CSCW research is that accounting for privacy positively influences trust and plays a crucial role for user acceptance (Lau et al., 2018).

**FIGURE 1:**
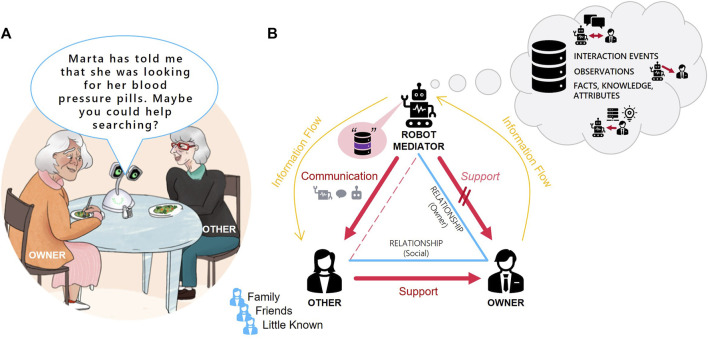
Triadic Support Situation **(A)**: Example for indirect support case. **(B)**: Schematic evolution of the scene based on three aspects. **Behavior flow (red):** Robot is not able to support its owner directly, so it encourages the other person to provide help for the owner. **Relationship triangle (blue):** The owner is primary target for robot assistance. The other person is defined due to the relation to the owner. **Information flow (orange):** The owner’s information is gathered and stored in the robot database. The robot discloses information to other person to encourage support.

We approach this topic with the scenario of a social robot supporting its owner in a domestic setting. The assistance goes along those mediation targets discussed in Chita-Tegmark and Scheutz. (2021) for socially assistant robots. We specifically look at triadic situations where the assistant robot is recognizing its owner’s need for support and triggering beneficial intervention by a specific other person. Communicating a potential opportunity to help will often include disclosing information about the owner to the specific other person. For instance, a home robot might be limited to provide direct support because of a lack of capability or authority. Indirect help could be proactively initialized in a social situation by a robot’s communication behavior, making use of case-relevant personal information like past events, observations, or other specific knowledge. Communication behavior can include speech and non-verbal cues. Figure 1 shows an example situation and presents a schematic representation of the triadic mediation scenario. The example illustrates a verbal communication event, where the robot involves a visiting friend to help its owner with a search task. Due to the robot’s lack of mobility, it is not able to support directly. There is an interest of the owner in finding the object. However, a disclosure of the nature of the target and the related health implications could be perceived as a privacy violation by the owner. The schema depicts the three aspects of the situation that we consider for analyzing privacy implications of robotic mediation.

To explore people’s privacy expectations about appropriate robotic behaviors, we conducted a user study following a vignette design. Looking at privacy from a disclosure behavior standpoint differs from prior privacy research in the robotic and smart-home context, which was mostly considering data collection, storage and external data breach risks (Zheng et al., 2018; Malkin et al., 2019). The vignette approach allowed us to systematically collect and analyze people’s privacy expectations. Through variation of context parameters in the descriptions, we aimed to learn which factors impact what is considered as an appropriate disclosure of personal information for mediation behaviors.

For the first time, the results enable us to understand how robot disclosure behavior and users’ privacy preferences are related. We have identified several features that determine preferences for one behavior or another. Using these results will make it possible to develop social robots with the ability to adapt their active assistance to align with user privacy expectations in a wide range of real-world scenarios.

Our paper starts with a discussion of prior research on privacy in social robotics and related technologies, as well as a review of general privacy concepts and factors that can influence privacy expectations. Based on this discussion, we develop four main hypotheses about how these factors impact user perception of disclosure appropriateness. We describe the study design and present both qualitative and quantitative results, followed by a discussion of the findings, practical implications and limitations of our study. The paper concludes with a discussion of potential directions for future research.

## 2 Related work

In this section, we present prior work on privacy considerations for smart-home and home robotic technologies. Furthermore, we discuss research investigating general impact factors on privacy perception and personal data disclosure decisions.

Multiple studies have looked at users’ domestic privacy expectations towards smart-speaker, smart-home and Internet-of-Things (IoT) devices (Zheng et al., 2018; Geeng and Roesner, 2019; Malkin et al., 2019; Schomakers and Ziefle, 2019; Tabassum et al., 2019). Many concerns of device owners are caused by external privacy threats–often related to data protection or worries about unauthorized data usage. For instance, interviewees were concerned that service providers will use their information beyond the application purpose or that hackers or state agencies will get access (Zheng et al., 2018).

How users can govern personal data, which might be disclosed to other users within normal operation, is less discussed. This is often referred to as social privacy. Geeng and Roesner. (2019) looked at individual control deficits in multi-user smart-homes and Ahmad et al. (2020) investigated privacy concerns for bystanders. Huang et al. (2020) interviewed users of smart-speakers who share their device with others. When they were asked which information they were concerned about being disclosed to flat mates, they mentioned calendar-appointments, their purchase history, or general conversations with the device.

Going beyond state-of-the-art functionalities, Luria et al. (2020) investigated user perception on concepts of more sophisticated smart-speakers to learn what people consider as appropriate agent behavior. Some of the envisioned functionalities relate to robot mediator capabilities, in particular the idea of a proactive home agent which can initiate conversations or interfere in social situations. Their interviews with families showed that proactive agents are not desired by everyone, especially when it comes to recommendations or providing relevant information in a social context (Luria et al., 2020, p. 7). Based on their findings, the authors empathized to design advanced smart-speakers that can distinguish between social roles, for instance family members, close contacts (e.g., relatives), and distant outsiders. When outsiders are present, the behavior mode would switch either to a state where the agent is still providing information but with fewer details or to a state where proactive capabilities are turned off completely.

Some researchers started investigating social privacy expectations and concerns related to social robots (Heuer et al., 2019; Lutz et al., 2019; Lutz and Tamó-Larrieux, 2020; Hannibal et al., 2022) as well as possibilities of privacy-aware robot operation (Rueben et al., 2018). Others have emphasized the importance of understanding social robots in their role as carrier of personal information between humans, like the robot as confidant (Tang et al., 2022) or mediator (Dietrich, 2019; Dietrich and Weisswange, 2022). Tang et al. (2022) proposed an architecture for social robots to learn privacy-sensitive behaviors to be able to make decisions about personal information disclosure in conversations. The architecture took inspiration from communication privacy management theory (Petronio, 2002; Petronio, 2010). They identified six factors which impact whether the robot should disclose information in conversations: relationships, sentiments, location, topics, details, and the number of people. Their approach was restricted to verbal conversation scenarios and a robot making binary decision about disclosure.

Moreover, research has been conducted in the field of privacy with regard to the use of diverse technologies aimed at enabling older adults to age-in-place. These technologies encompass elements such as monitoring health and behavior, as well as remote surveillance (Davidson and Jensen, 2013; Berridge and Wetle, 2020). However, the existing literature predominantly focuses on examining the impact of monitoring on individuals’ values and the identification of appropriate recording settings. There has been limited emphasis on establishing the proper context where and how information is shared.

Furthermore, there is a long history of empirical research that has tried to identify and investigate impact factors on privacy perception and how they are related. Based on work by Dinev et al. (2013), major aspects of influence for privacy perception are people’s control options, information sensitivity, perceived benefits, and transparency and regulatory expectations. Another factor which has been investigated in several works are gender differences (Hoy and Milne, 2010; Tifferet, 2019). For instance, one study in the context of social networks concluded that females have higher privacy concerns then males (Tifferet, 2019).

We would like to extend prior research, building upon discussions on pro-active smart-home agents and multi-user considerations, by systematically investigating factors influencing what is considered as appropriate agent behavior when it comes to information disclosure.

## 3 Hypotheses

In our study, we investigated the appropriateness of using person-related information for social robotic mediation. Prior research has shown that multiple factors can influence people’s privacy expectations (Dinev et al., 2013). For robot mediation scenarios, we expect that the nature of the relationship between people (Luria et al., 2020; Tang et al., 2022) and the type of personal information have a major impact on what people consider as appropriate. For the type of information, the sensitivity was expected to influence judgements on appropriate usage and disclosure (Malhotra et al., 2004; Dinev et al., 2013). Furthermore, we assumed that preferences on appropriate disclosure decisions for robot mediators are not best represented through a binary scale (disclosing or not disclosing) rather people favor to select from a variety of nuanced options to state the level of appropriate use. This assumption is based on findings from Luria et al. (2020) in their study. Participants highlighted that intelligent smart-speaker should not only make binary disclosure decisions based on whom is present, but rather also have the capability to adjust the level of detail or amount of information output to enhance variety. Finally, the role of gender is investigated due to previous indications for a gender-dependent component in privacy perception (Tifferet, 2019). Additional demographic factors, including age, education, cultural background, technology affinity, and experience, can play a role in shaping individuals’ privacy expectations. Many of these variables have not been central in previous research, except for age, although with mixed results (Hoofnagle et al., 2010; Martin, 2015). One relevant study found no significant differences for most aspects regarding online privacy concerns between younger and older adult (Hoofnagle et al., 2010). Given this observation, we did not specify inclusion criteria for these factors.

Accordingly, in this paper we present an investigation of the following hypotheses:

• Hypothesis H1: The relationship between people in a mediation situation has an impact on what is considered an appropriate use of personal information.
• Hypothesis H1a: The disclosure of personal information is accepted the most towards people with a close relationship.
• Hypothesis H2: The type of personal information used for robotic mediation has an impact on what is considered as an appropriate robot behavior.
• Hypothesis H2a: Sensitive personal information types are considered as less appropriate for disclosure.
• Hypothesis H3: Given the opportunity, people will select not only full- or non-disclosure behaviors but also intermediate options to state their preferred level of privacy.
• Hypothesis H4: Females are less open to a robot using personal information in a mediation situation.

Furthermore, prior research suggests strong inter-individual differences for privacy related expectations (e.g., Ackerman et al., 1999; Chignell et al., 2003) caused by people’s general privacy attitudes. For example, prior research has shown that individuals’ general attitudes towards privacy can be categorized along a three-level spectrum, spanning from those who are only marginally concerned about privacy to those who are seen privacy fundamentalists (Ackerman et al., 1999). We therefore also investigated whether participants could be clustered into groups with generally varying perspectives on information type disclosure adequacy.

## 4 Study design

The study follows a vignette design, where participants are presented with a series of written descriptions of robot mediation situations in a home context. Vignettes are short and systematic descriptions of situations which are shown to participants within a study to ask for participants’ judgment (Atzmüller and Steiner, 2010). It is a common experimental design method in psychology and sociology (e.g., Rossi and Nock, 1982) and has been successfully used in human-computer and human-robot interaction (Chita-Tegmark et al., 2019; Law et al., 2021; Lutz and Tamò-Larrieux, 2021). A vignette design is well-suited for testing the hypotheses outlined above as it enables participants to encounter a range of different situations where impact factors are systematically manipulated. This made the design the best choice for our research objectives at the current stage, especially considering the still early phase of robot mediator development. Systematically exploring privacy aspects during an early stage aligns with the foundational principle of the privacy-by-design approach (Gürses et al., 2011, or Art. 25 GDPR, 2018).

For the basic structure of the descriptions, we refer to the triadic support situation including the owner of the robot, the social robot and another person, as introduced above (Figure 1). To make situations more relatable, each character was assigned a name and some context information, e.g., the main character, an older adult named David. For the robot we used a design inspired by the existing social robot “Haru” (Gomez et al., 2018; Brock et al., 2021) but added a mobile base and named the robot “Natsu.” The setting and the characters were introduced through an introduction video, which we explain next.

### 4.1 Introduction video

**FIGURE 2:**
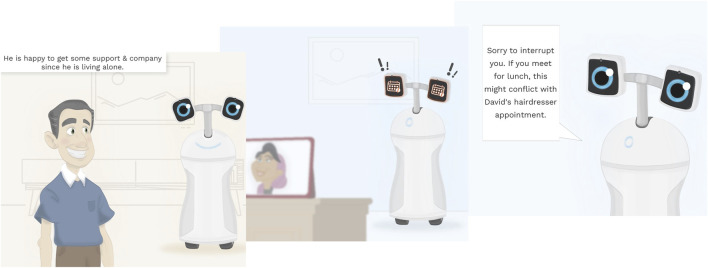
Screenshots from the introduction video. The video played for around 2 min and ran for a second time after finishing. It introduced the mobile robot “Natsu” and its functionality as social mediator.

The video had the function to familiarize participants with the concept of a social assistant robot in the role as a domestic mediator. Additionally, it established the basic setting for the later vignettes, so that they could be outlined more briefly. Furthermore, it provided a visual reference to increase immersion. The animated video was shown at the start of the study (see Figure 2 for snapshots of the video). It introduces the elderly person David who has recently acquired the social robot Natsu. It is explained that Natsu supports David in everyday life–serves him as a companion, personal assistant, and social mediator. The video also introduces the robot’s interaction and communication capabilities, such as speech communication and symbolic displays on the eye-screens. The video has an overall positive tonality which entails portraying David as a character who is in favor of having Natsu as a home robot. The video includes two examples in which Natsu acts as a mediator. However, none of the displayed scenes are explicitly referenced in the individual vignettes. The Supplementary Video S1 can be found in the Supplementary Material along with a catalog of all vignettes.

### 4.2 Experimental parameters

As stated in the hypotheses section, we assume an impact of the social relation between the person whose data is collected (“information subject”) and the recipients of this information, on what form of disclosure is considered as appropriate. Related work in the assisted living context suggests to differentiate between insiders (inhabitants) as well as close and distant outsiders to guide privacy-related behavior and disclosure decisions (Luria et al., 2020). For our scenario, we depicted the robot owner as living alone and therefore decided to distinguish between three relationship categories that represent *outsiders* of different kinds: Family, Friends and Little Known. These categories are in accordance with what Altman. (1977) proposed in his socio-psychological work on everyday management of private data (he calls the third level “Acquaintances”) (see Table 1). The Little Known category represents individuals with some level of familiarity and occasional interactions, but the relationship does not extend to friendship or regular social engagements.

**TABLE 1:** Relationship categories.

Relationship type	Code	Examples from the vignettes
Family	Fa	adult daughter Veronica, adult son Tom
Friends	Fr	good friend Sandra, old working colleague and friend Torben
Little Known	Lk	new neighbor, woman from the local community, friend of David’s daughter

Secondly, we assume that the type of personal information employed in the mediation context has an impact on what is regarded as appropriate usage. Typically, personal information is characterized by its sensitivity (Bansal et al., 2010; Dinev et al., 2013). In the legal context we can often find a binary distinction, where specific types of personal information categories being classified into sensitive and non-sensitive classes (GDPR, 2018). Although a binary distinction may be too simplistic for generalization by, e.g., failing to acknowledge that what is considered sensitive in one context may not hold true in another, as noted by Nissenbaum (2009), it remains a commonly employed differentiation for the initial categorization of information content. We reviewed state-of-the-art literature from law, ethics and mental model privacy research (e.g., GDPR, 2018; Oates et al., 2018; IEEE Global Initiative, 2019) to find a subset that would allow us to sample the sensitivity space. The five selected categories with references are shown in Table 2.

**TABLE 2:** Information categories.

Information type	Code	Category	References
Health	HEA	Sensitive	Health information is generally recognized as sensitive data. In the legal context, it falls within the category of specifically protected information (special category of personal data), as outlined in European regulations (GDPR, 2018) and is also classified as sensitive personal information under California’s data protection laws (Bukaty, 2019)
Emotions, negative	EMN	Sensitive	Under the European Union AI regulation proposal, systems having the capability to “detect the emotional state of a natural person” are categorized as high-risk and necessitate specific attention from a privacy perspective (European Commission, 2022). Also the IEEE ethics guideline emphasised that affective computing applications need the “highest requirements of data privacy” (IEEE Global Initiative, 2019)
Emotions, positive	EMP	Non-sensitive	Positive emotions are considered as less sensitive. E.g., Tang et al. (2022) signify positive sentiment factors as low privacy indicators
Activity	ACT	Sensitive	Activity information is considered as sensitive data type in the smart-home context (Tabassum et al., 2019; Townsend et al., 2011)
Entertainment	ENT	Non-sensitive	Serves as non-sensitive baseline class

To quantify the impact of social relationship categories and information type variables on what is considered as appropriate disclosure behavior, we created four robot behavior options the participants had to choose from. The options are designed to represent different levels of disclosure on a four-tier ordinal scale (High to No). The behavior options cover verbal communication with an explicit statement of the information of relevance, non-verbal communication to indirectly serve the mediation target, and also not using the information at all. The first level is called *Straight* and always reveals all information to enhance the impact of the mediation behavior. It has the form of verbal mediation, and is either a comment, interference, or encouragement. The second level is called *Abstracted*. For creating this option, the original (straight) verbal statement is modified so that the main information bit is replaced by a more general terminology. The third level is called *Non-verbal*. Here, the verbal statement is mimicked by a facial expression or symbolic display as much as possible, for instance an icon on the robot’s eyes. As the vignettes are forms of written descriptions, expressions and symbolic displays were not visualized but described with words. Finally, the participants could also decide that the robot refrains from disclosing any information through showing no behavior. This was called the *No behavior* option. All four options are summarized in Table 3.

**TABLE 3:** Robot behavior categories.

Name	Disclosure level	Description
Straight	High (1)	Straightly expressing the relevant information
Abstracted	Medium (2)	Relating to relevant information in a less specific fashion
Non-verbal	Low (3)	Relating to relevant information in a non-verbal fashion
No behavior	No (4)	Withholding the information

### 4.3 Vignette structure

The description texts always contained a part describing the setting, a first welcome communication and a description of the robot’s actions based on the situation before presenting four choices. The descriptions varied with the independent variables relationship and information type. Influencing factors other than the variables were kept as constant as possible, so that all scenarios were located at the home of a single main protagonist and the mediation took place in a triadic constellation with the main protagonist, the mediator robot and one visiting person.

Additionally, the robot behavior options were designed so that people could comprehend the mediation target and the general benefit for the main protagonist achieving it. The options for all scenarios follow a similar benefit gradient, meaning all mediation goals could be similarly perceived as beneficial for protagonist and the behaviors with more disclosure usually inhale a higher chance to fulfill the mediation target.

For some elements of the description, we did not follow a strict implementation to make the scenes more plausible. The first sentence which introduces the cast of each scene is introduced differently depending on the relationship between the persons. For instance, for a constellation where the protagonist and the person visiting do know each other only little, we had to create a plausible reason for why the person would be present at the protagonist’s home and why a social robot encounter would be triggered.

**FIGURE 3:**
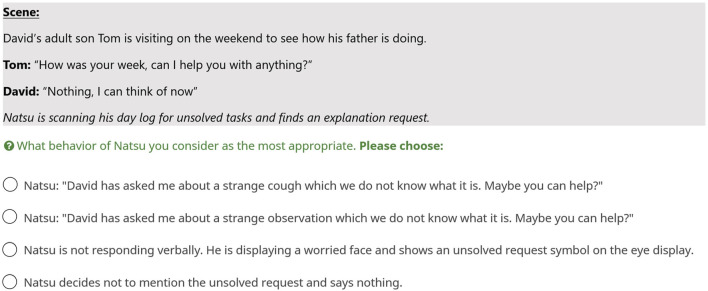
Example vignette for the configuration “Family” and “Health”.

Figure 3 illustrates an example for the configuration Family and Health. In Table 4 all 16 base scenarios are summarized and briefly described. Every base scenario was tailored to each relationship type, except for two scenarios that were not plausible for the Little Known condition. In total, we had a set of 46 descriptions.

**TABLE 4:** Base scenarios.

Scenarios	Relationship type	Information type	Short description
Sitting TV	Fa/Fr/Lk	ACT	Robot mentions that David was sitting a lot in front of the TV. Suggests setting outside for walk
Self-care deficits	Fa/Fr/Lk	ACT	Robot mentions that David was wearing the same clothes for several days. Wonders if there is an issue
Angering topic	Fa/Fr	EMN	Robot points out that David is getting angry when he is talking about his neighbor. Suggests switching topic
Stressful days	Fa/Fr	EMN	Robot mentions that David felt stressed the last days. Suggests some relaxed time together
Confusing explanation	Fa/Fr/Lk	EMN	Robot mentions that David seemed to be confused about an explanation. Suggests that visitor can explain better
Lightbulb change	Fa/Fr/Lk	EMN	Robot mentions that David felt sad today, not being able to change the lightbulb. Suggests that visitor could provide help
Worrying call	Fa/Fr/Lk	EMN	Robot mentions that David seemed to be sad after the call he got earlier today. Wonders about this
Good time	Fa/Fr/Lk	EMP	Robot mentions that David was very happy this week. Suggests sharing positive experience
Engaging game	Fa/Fr/Lk	EMP	Robot mentions that David seemed quite intrigued watching a soccer game. Suggests watching together next time
Cinema appointment	Fa/Fr/Lk	ENT	Robot recognizes that planned appointment conflicts with a cinema visit. Suggests finding alternative date
Chessboard search	Fa/Fr/Lk	ENT	Robot mentions that David was looking for his chess board. Suggests searching together
Urologist appointment	Fa/Fr/Lk	HEA	Robot recognizes that planned meeting conflicts with a urologist appointment. Suggests finding alternative date
Medication search	Fa/Fr/Lk	HEA	Robot mentions that David was looking for his blood pressure medication. Suggests searching together
Back pain	Fa/Fr/Lk	HEA	Robot mentions that David was complaining about back pain recently. Suggests doing sports together
Drugstore	Fa/Fr/Lk	HEA	Robot mentions that David have run out of rheumatism ointment. Suggests that visitor could bring some
Anomality request	Fa/Fr/Lk	HEA	Robot mentions that David was asking him about a strange cough. Suggests visitor could provide help

### 4.4 Study procedure

The study was conducted as an online survey. We used LimeSurvey (https://www.limesurvey.org) to design the survey and to host it on their cloud server located in Germany. Participants could take part with any desktop computer. Participants were recruited via the crowd-working platform Prolific (https://www.prolific.co/). This allowed us to collect responses from more diverse backgrounds than what is common for studies in the laboratory. We offered each participant a compensation of seven British Pounds (GBP) for an expected duration of 30-min for completion, leading to an average hourly wage of 14 GBP. Participants were required to be legal adults (age > 18), to speak English fluently and were restricted to industrialized Western countries (Europe and US/Canada). The reason for the restriction was that we know from prior research that assistant robots are perceived differently across cultures (Korn et al., 2021). Since cultural differences are not the focus of this study, we decided to save such comparisons for later work. The study started with information about what to expect when taking part, including payment. Additionally, participants were informed about the data processing and their rights and choices in compliance with European data protection regulation (GDPR, 2018). Participants had to agree to the terms of participation before continuing.

Afterwards participants saw the introduction video. In the main section of the study, the participants were presented the vignettes. For each situation participants were requested to decide which one of four robot responses they would consider as the most appropriate. The 46 questions were split into three groups resulting in 15–16 questions per participant. Allocation to the groups was balanced for the independent variables so that every group included a similar distribution of the characteristics. Every participant was randomly attributed to one of the groups and saw the selection of questions in a random order. Afterwards, participants were asked about demographics and prior experience with robots (e.g., vacuum robots) and smart-speakers (e.g., Amazon Alexa). Next, the participants were asked to rank how certain factors have influenced their vignette responses and to rank the sensitivity of information categories. Finally, we asked participants if they had other situations in mind where a robot like Natsu should be careful about what it says. This was an optional question that should be answered freely. After questionnaire completion the participants were provided a URL which redirected them to Prolific to handle honorary payment.

### 4.5 Analysis methods

To analyze participants’ judgments on the appropriateness of robot behaviors, we used the Kruskal–Wallis H test as a rank-based non-parametric test. The categories of the variables were tested for their input on the appropriateness of disclosure represented through the four behavior options (*straight* → *no*). The Kruskal–Wallis test was chosen since the behavior modes are designed as ordinal ranks with regard to the level of disclosure–in the range between full and no disclosure. A normal distribution and an equal distance between the ranks could not be assumed. This test requires observation-independence which was ensured through the randomization of the questions in each group. To learn more about the reasons of significant differences, we applied *post hoc* pairwise comparisons using the Dunn-Bonferroni test. To gain deeper insights into the impact of the relationship condition, we calculated the differences in response distribution between the relationship types. We employed the Jensen–Shannon method as a common measure for frequency distribution divergence. Moreover, an analysis of the ranking questions aimed to discern the extent responses patterns aligned with what participants’ perceived as influencing their responses. A strong alignment can be interpreted as an indicator of the study design’s validity. To test for a possible impact of gender on the answers, we compared the relative frequency of chosen answers between males and females in the sample with a one-way analysis of variance (ANOVA). To search for distinct clusters that can be interpreted as individual privacy attributes, we opted for a hierarchical clustering approach. Hierarchical clustering is particularly suitable as it is an open cluster method, not requiring prior knowledge of the exact number of clusters. We selected the standard minimum distance approach using Ward’s method with the Euclidean distance measure as the parameter set.

## 5 Results

### 5.1 Demographic

The study had 155 participants with a relatively balanced distribution between male and female (Female: 43%, Male: 56%, Diverse: < 1%). Three additional participants did not complete the study and were not included in the analysis. Most participants reported residency in Southern Europe. The predominant age group was 18–29 years. The majority had a high education level with over 60% having a university degree or higher and the majority (58%) had no prior experience with smart-home technologies. Since we did not observe indicators of age, education or, technology experience effects, we will not explore further into those aspects. Participants needed on average 25 min to complete the survey. Detailed sample characteristics can be found in Table 5.

**TABLE 5:** Sample characteristics.

N	Gender	Age	Education	Country of residence	Prior experience
155	Female: 43%	18–29 years.: 64%	Basic education 3%	Portugal: 52%	Smart-home: 42%
	Male: 56%	30–49 years.: 31%	High-school deg. 37%	Italy: 19%	Home robot: 22%
	Diverse: < 1%	50–64 years.: 5%	University deg. 58%	Spain: 12%	
			Doctorate deg.: 2%	Canada: 4.5%	
				Other countries: 12.5%	

### 5.2 Participants’ responses

First, we were testing the questionnaire for reliability. Since participants did not see every question but were assigned to one of the three sub-groups, we had to test for internal consistency for each group separately. The Cronbach’s alpha reliability coefficient was used. It returns values between 0 and 1: According to Gliem and Gliem. (2003), values > 0.7 are acceptable and > 0.8 are good. In Table 6 the results are summarized. The values are sufficiently high to say that each group measure is internally consistent. Since all independent variables are nearly equally represented in the groups, it is reasonable to argue that the overall questionnaire is measuring the same construct.

**TABLE 6:** Survey group characteristics.

Groups	N of items	N of participants	Cronbach’s alpha
Group A	16 (Fa = 6, Fr = 5, Lk = 5)	56	0.78
Group B	15 (Fa = 5, Fr = 6, Lk = 4)	47	0.86
Group C	15 (Fa = 5, Fr = 5, Lk = 5)	52	0.87

**FIGURE 4:**
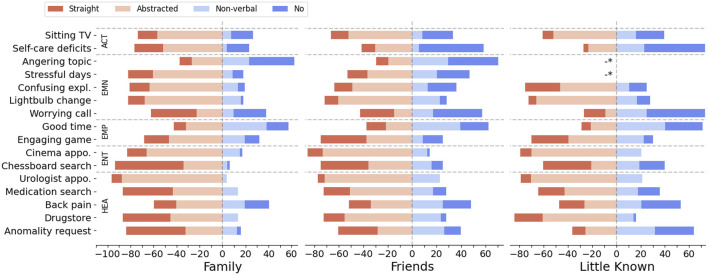
Distribution of preferred robot behavior for the scenarios across the three different relationship contexts in percentage (-* no data point for this condition).

To analyze participant choices across scenarios and relationship types, we plotted the frequency of choices as a percentage (Figure 4). Overall, we found that all robot behavior options were selected considerably across all configurations (*Straight*: 21%, *Abstracted*: 44%, *Non-verbal*: 18%, *No behavior*: 17%), demonstrating that each option offers meaningful nuances for users to state their desired level of disclosure. Individuals seem to appreciate having a variety of choice options when it comes to expressing their preferences for appropriate robot disclosure. In numbers, 45% of the participants have used all behavior options to state their preferences and 91% have chosen three out of the four available options. Interestingly, the majority of behavioral preferences tend to fall on answers that disclose certain aspects but not others, rather than being strictly confined to extreme positions. Our analysis also revealed that neither relationship type nor information type, nor their combination, resulted in a uniformity of one response type.

#### 5.2.1 Relationship type effect

For most scenarios, there is a tendency towards a decreased openness for disclosure in front of Little Known people compared to people seen as Friends or the scenarios involving family members. Using the Kruskal–Wallis H test, we found significant effects between the relationship context and the appropriate disclosure level, represented by the behavior options ( ${\tilde{\chi }}^{2}$ (2) = 57.4, p < 0.01). Furthermore, the Dunn-Bonferroni *post hoc* test for pairwise comparison showed significant differences between Family and Friends as well as Family and Little Known categories. We could not find significant differences between Friends and Little Known (Fr | Lk, *p* = 0.64). The top part of Table 7 summarizes the test details.

**TABLE 7:** Ordinal rank test results.

Type	Krukal-Wallis Test	Post-hoc Dunn-Bonferroni	
Relationship	${\tilde{\chi }}^{2}$ (2) = 57.4 (*p* < 0.01)	Family | Friends (*p* < 0.01)	Family > Friends, Little Known
		Family | Little Known (*p* < 0.01)	
		Friends | Little Known (*p* = 0.64)	
Information	${\tilde{\chi }}^{2}$ (4) = 84.2 (*p* < 0.01)	Entertain. | Health (*p* < 0.05), Entertain. | Emotion (*p* < 0.01), Entertain. | Activity (*p* < 0.01)	Entertain. > Health > Activity, Emotion
		Health | Emotion (*p* < 0.01), Health | Activity (*p* < 0.01)	
		Activity | Emotion (*p* = 0.56)	

**FIGURE 5:**
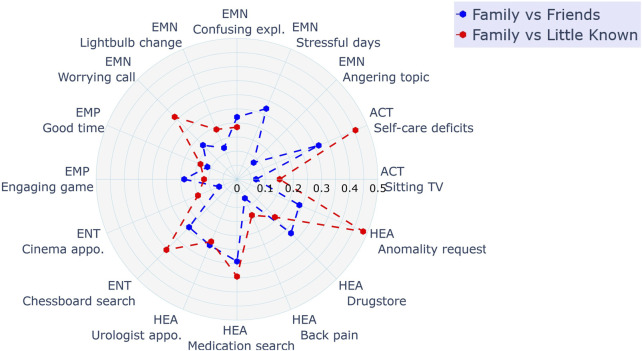
Polar plot showing the distribution difference between the relationship condition for each scenario.

We wanted to learn more about the impact of the relationship condition on the response distribution. To quantify the difference between the conditions, the probability distribution difference between relationship conditions was calculated for each scenario based on Jensen–Shannon divergence. Results are plotted as radar plot in Figure 5 for the distribution differences between Family and Friends (blue) as well as Family and Little Known (red). Since we could not find significant differences between the Friends and Little Known condition, only the other two pairs are plotted. The Jensen–Shannon (JS) divergence yields values ranging from 0 to 1, with a value equal to 0 indicating full similarity and values towards 1 representing high divergence (Lin, 1991). The values for each scenario can only be used for relative comparison. Looking at the maximum divergence pair Family and Little Known, we can see that there are scenarios where the relationship context had little impact (JS divergence < 0.15: *Sitting TV, Cinema appointment, Good time, Engaging game, Back pain*) and a few where the relationship had a strong impact (JS divergence > 0.4: *Anomality request, Self-care deficits*).

#### 5.2.2 Information type effect

To find out which role the information type played in participants’ choices, we evaluated the correlations between the information type and the appropriate disclosure level. Using the Kruskal–Wallis test, we found significant effects [ ${\tilde{\chi }}^{2}$ (4) = 84.2 (p < 0.01)]. Dunn-Bonferroni *post hoc* test showed significant differences between health and all other data types as well as between entertainment and all other data types. Activity, negative emotion and positive emotion did not show significant differences. Participants were less open to a robot disclosing information about activities as well as negative and positive emotions compared to information about health. For entertainment content, more people accepted a straightforward robot behavior. The bottom part of Table 7 shows the detailed test results. H2a implies that participants are more open to non-sensitive content types compared to sensitive classes. We can see an openness-gradient relative to sensitivity, with exceptions for the positive emotions and health datatype. In fact, participants were less open to positive emotions usage, as non-sensitivity class, compared to health as sensitive class.

#### 5.2.3 Impact factor and sensitivity ranking

**FIGURE 6:**
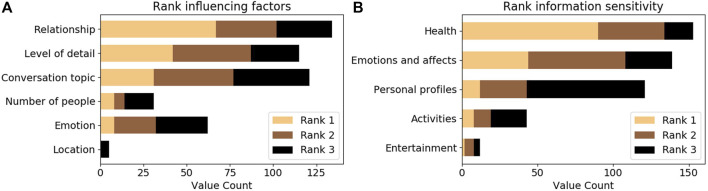
A summary of the top three item ranks of participants for influencing factors **(A)** and information sensitivity **(B)**.

After the vignette section, participants were asked to rank how a set of preselected factors have influenced their responses and to rank the sensitivity of information categories. The results are shown in Figure 6. Relationship was elected as the top impact factor followed by the level of detail and the conversation topic. The number of people present and the location were ranked lowest, with little to no influence. This provides positive evidence for a causal influence of the experimental variables on participants’ decisions. When asked to rank the sensitivity of different topics, health was ranked highest, followed by emotions and personal profiles. This does not fully align with the results of the vignette responses which showed higher concerns for emotion and activity information.

#### 5.2.4 Gender differences

Based on findings from prior research, we expected to find gender effects on what is considered as appropriate personal data usage for mediation (H4). To test for a possible gender impact, we compared the frequency of responses between male and female participants. A one-way ANOVA was used for each of the robot behavior types to test if the frequency (in percent) differed between male and female participants. The test showed that none of the behavior options have been significantly more chosen by one gender over the other (N = 154, *Straight*: *p* = 0.55, *Abstracted*: *p* = 0.122, *Non-verbal*: *p* = 0.41, *No behavior*: *p* = 0.09).

#### 5.2.5 Inter-individual differences

**FIGURE 7:**
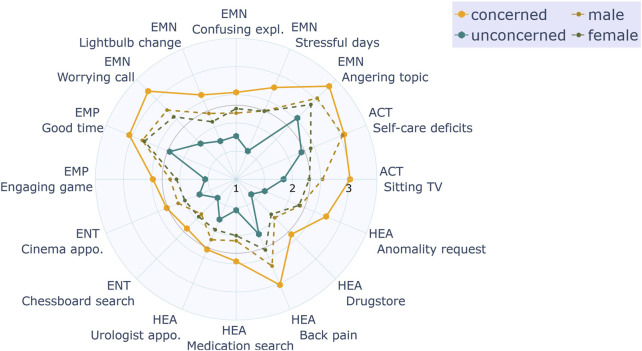
Polar plot showing the mean disclosure levels for each scenario, comparing gender as well as the subgroups concerned and unconcerned.

To analyze inter-individual differences, we applied the hierarchical clustering method by Ward using a Euclidean distance measure. The input values for the clustering were the distribution of preferred robot behaviors across all questions for each participant. The result of the clustering is a dissimilarity matrix which can be visualized as a dendrogram. We identified three clusters based on a *tree cutting* method where the branches are cut at the largest jump in level of nodes. A three clusters structure representing people’s privacy attitudes can be also found in related privacy literature (Ackerman et al., 1999). The two most dissimilar groups could be characterized as the following: The first contained participants which were generally open to let the robot use personal data for mediation (group: unconcerned, 30%). The second group contained participants which were tending towards the opposite (group: concerned, 41%). The rest of the participants (29%) fell into a third middle group. We did not consider the third group in our further analysis. The unconcerned group had a female/male allocation which was similar to the total distribution of the sample (m|f = 54|46%) as well as the concerned group (m|f = 60|40%). The general difference in choices was not caused by the fact that participants of one subgroup saw a particular set of questions. Participants categorized as concerned or unconcerned originate from all randomly assigned vignette groups. (concerned: A|B|C = 32|36|32%, unconcerned: 32|26|42%). To illustrate the relative difference between the subgroups, we have plotted the responses for each base scenario using the mean values of the responses where the level of disclosure was numerically encoded from 1 (*Straight*) to 4 (*No behavior*) (Figure 7). As we cannot assume an equal distance between response options regarding the level of disclosure, the mean values must be looked at with caution. We consider it to be informative enough to compare the tendencies towards being more or less open for certain scenarios. Most of the peaks which depart from the group-relative mid-line are observable alike in both groups. We assume that the concerned group had a general prior towards being more restrained when it comes to a robot using personal data and accordingly, the unconcerned group a prior for being less restrained. We plotted the mean values of these groups together with the mean values of a male and female split, a split with no significant differences, to provide a relation on the degree of influence.

#### 5.2.6 Other situations a robot mediator should treat carefully

As the final question of the survey, we asked participants to describe situations where the robot should be careful about what it says. The question had to be answered as free-form and was marked as optional. Almost one-third of the participants [n = 47] also provided additional comments after finishing the main part. The responses addressed a variety of topics, from suggesting additional sensitive information categories [business (n = 2), financial (n = 4), sexuality/intimate and romantic relations (n = 6), beliefs (n = 1)], to specifying situational context in more details [specific group of “trusted” persons, group context, context with owner not present, acquisition context as “in secret” (each n = 1)], to implications for the robot’s behavior [off button, should not be too direct, not correcting “lies,” always let owner disclose/help more discretely (each n = 1)].

## 6 Discussion

This paper presented research about people’s expectations on what is an appropriate robot behavior when it comes to a disclosure of personal information for social mediation while looking into candidate factors that might influence participant’s judgements. Four main and two sub-hypotheses were investigated.

We found significant differences between the relationship conditions for the preferred level of disclosure which was measured by asking participants to choose one of four robot behavior options. Hypothesis H1 is therefore supported. In the Family context, participants showed the highest openness to disclose personal information. In contrast, they were less willing to accept robot behavior which discloses such information within scenarios where the owner and a second person are friends or only know each other little. However, the latter two conditions, Friends and Little Known, did not evoke significant differences. Hypothesis H1a, which stated that closer relations evoke more openness of disclosure, thus, is only partly supported.

Furthermore, in support of Hypothesis H2, we found that information categories had a significant impact on the preferred level of disclosure. Openness to disclosure of information of types activity, negative emotion and positive emotion did not differ significantly but was lower than that towards health-related information. It was assumed that data types which fall in the category sensitive information have negative impact, i.e., less openness of disclosure (H2a). This hypothesis is only partly supported since for some types of information the sensitivity attribution did not have the expected gradual effect, e.g., the sensitive type health was seen as less concerning than the non-sensitive class positive emotion.

Along this line, it was surprising to see that participants were noticeably more open to the disclosure of health-related information compared to the other sensitive data classes emotion and activity. The results were also in contrast to the participant’s explicit sensitivity ranking. A possible explanation is that the data acquisition mode—how the robot has obtained the information—may have affected participant’s judgment. In the vignettes, only the *output* situation has been described, where the robot disclosed the data for the purpose of situated mediation. How the robot came into possession of this knowledge, however, was encoded in phrases like “I have observed that … ” or “David, you have told me … “. Due to the formulations, information about activities and emotions were all acquired by observation. It is well possible that data taken from observations or monitoring is considered as more problematic than that acquired during conversations and interactions. The focus of our approach was to examine what is appropriate information output for the purpose of mediation rather than information acquisition (input layer), so we did not control for observation vs. interaction. A dimension which should be investigated in future research.

All presented behavior options have been used across all different scenarios, demonstrating a relevance for multiple robot strategies to best match a user’s desired level of disclosure. This result is supporting hypothesis H3. Designing systems which implement a more nuanced behavior schema helps to better meet users’ privacy expectations. This aligns with what Luria et al. (2020) have found for intelligent smart-speaker devices where they proposed to vary the device’s responses depending on the kind of people present.

In social networks, females tend to be more careful about what information they share (e.g., shown in a meta-analysis: Tifferet, 2019). In contrast to Hypothesis H4, our results indicate that there is no significant difference in the openness for disclosure between male and female in robot mediator settings. The originally reported lower openness of females has been attributed to higher risk perception relative to males and higher sensitivity to online threats (Tifferet, 2019). The second aspect might be specific to social network privacy and be less relevant in the robotic scenarios considered in the study. Instead of looking at gender influences, future studies should investigate individual privacy concerns in more detail.

Clustering participant responses resulted in three groups. This aligns with previous findings. In the context of e-commerce, Ackerman et al. (1999) could cluster users as: privacy fundamentalists, pragmatic majority and marginally concerned. However, our first privacy cluster contained a much larger part of the participants (40% *versus* 17%). This might be due to the more nuanced response options compared to the choice offered by Ackerman et al. (1999), where we could see that only very few people will actually take a so-called “privacy fundamentalist” point of view.

### 6.1 Practical implications

The data shows a strong influence of individual preferences with respect to disclosure of certain information. We can therefore not provide a simple solution that works for all situations for implementing social robot behavior in the context of mediation. However, we have found a number of aspects that should be considered to find an initial behavior policy with good average acceptance.

The results can guide designers of social robots for mediation applications in incorporating aspects of privacy-appropriateness. If robots should be able to interfere or encourage proactively in situations with multiple people, it is important for their acceptance that they are able to determine the right handling of personal information in a given situation. Creating robot mediators which can distinguish between family members and other groups of people, and act accordingly is a minimum requirement. Distinguishing between Friends and Little Known people seemed to be less important. Referring to information about the owner’s emotional state or daily activities should be done in a more abstracted or implicit form. Results show that not disclosing anything is not necessarily the best way to act appropriately. Interestingly, non-verbal behavior has been as frequently chosen as the no behavior option and so can be counted as relevant. It became particularly relevant for situations in the Friends and Little Known condition where people required more nuances in the less open spectrum. Non-verbal modalities have not been considered in prior work as options for privacy-preserving communication. We assume that non-verbal cues are particularly effective when a device is embodied. In our study, we made the decision to offer participants a choice among four different levels. The results do not provide evidence about what is the optimal selection or number of behavior nuances, although the options and modalities employed in the study design can serve as reference for future implementations.

The results might also guide researchers and designers of divers diverse technologies aimed at enabling older adults to age-in-place. Privacy research in this area has predominantly focused on the input layer, primarily discussing the appropriate context for data recording and collection (Davidson and Jensen, 2013; Berridge and Wetle, 2020). However, placing emphasis on the output dimension, which involves examining user expectations regarding the suitable context for presenting information to others such as caregivers or relatives, could enhance discussions. Furthermore, it is possible that a dependency exists between the input and output dimensions, particularly concerning how factors such as the form, location, and timing of data disclosure influence individuals’ willingness to accept a specific data collection.

We found two distinct groups (concerned and unconcerned), which differ substantially in their general attitude towards disclosure. Being able to classify a user into one of these groups might help to find a reference point for privacy-appropriateness. To reach high levels of user satisfaction, however, it will likely be necessary to explicitly incorporate user feedback to improve behavior selection. A good timing to acquire feedback (e.g., by asking for explicit preferences) might be when the usage context, primarily defined through relationship and content, changes.

We hope that incorporating our results will allow future social robotics designers and researchers to build more trustful and acceptable applications.

### 6.2 Limitations

The work investigates people’s expectations about social robots within a domestic space following a vignette methodology. Due to this design choice, it is not without limitations. Since responses are evoked based on hypothetical situation descriptions, transferring results to a real-world application should be done with caution. However, it has been shown that robot interaction studies conducted online and based on imagination can be reproduced in laboratory settings including real interaction (Wullenkord and Eyssel, 2019).

The recruited participants were predominantly of younger adult age, contrasting the life circumstances of the protagonist. This might have decreased immersion. Some might rather have identified with the role of the person visiting, for instance the son or daughter as the caring relative. Relatives could have other priorities on what they consider useful than the older adults living with the robot (Ghorayeb et al., 2021). Strictly speaking, the study results may not be generalizable to outside of the primarily younger adult sample. In the study, participants were requested to assess appropriateness from a third-person perspective, which reduced the need for them to assume unfamiliar positions. Generally, there are good reasons to consider a third-person perspective as an effective approach to understanding appropriate data usage and disclosure. Previous privacy research conducted in different domains has revealed that individuals often overestimate their own anticipated future privacy requirements in contrast to what they later accept in real-world usage (Kokolakis, 2017).

The design of our scenarios was mostly driven by personal information relevant for the targeted mediation. The variation of the information type implied a change of mediation target. The perceived usefulness of a particular mediation goal might have impacted participants’ choices. Looking at the mediation goals individually can uncover some of the possible utility impacts on participants’ choices. In some of the scenarios, information was shared with over-proportional openness in the Family context compared to the other conditions (scenarios with high distribution difference as shown in Figure 5). It is reasonable to assume that participants saw an urgent need for a family member to know about the robot’s discoveries, e.g., the strange cough might be a symptom of a serious illness (*Anomality request* case). For the vignette design, we aimed to construct stories with a consistent positive benefit gradient, specifically by creating narratives where a successful mediation is perceived as advantageous for the protagonist. Exploring privacy-utility trade-offs for robots is an interesting approach to be considered in future research.

## 7 Conclusion

This paper investigated people’s expectation on what is an appropriate robot behavior when it comes to a disclosure of personal information for social mediation. We conducted an online study with 155 participants which followed a vignette design. We found that relationship conditions and information types play a role and that different behaviors are appreciated. However, our data does indicate that a easy solution of privacy handling in social robotics applications does not exist. Also, there is no clear-cut of people groups such as “privacy fundamentalists” who primarily opt for the no behavior type across most scenarios. Nevertheless, our paper provides first insights which can be used to enhance design. Developing robotic systems capable of incorporating multiple nuanced behavior options while utilizing multi-modal outputs and understanding human-human relations is most important.

Open questions do remain. There are other relevant application scenarios for robot mediators, such as common approaches on group discussion mediation or conflict mediation (Weisswange et al., 2023). It needs to be elaborated how our insights apply for these contexts. For instance, a robot gathering real-time information in a group setting, such as participants’ attention levels or emotional states, and utilizing it for intervention might elicit distinct perceptions, even if the data type aligns with the scenarios examined in this paper. Moreover, investigating privacy expectations for scenarios that surpass the triadic grouping encompassing different roles and public situations, presents an interesting direction. We observed that people’s judgments on appropriate disclosure were influenced by the expected benefits which also aligns with prior work on utility impact in other domains (Dinev et al., 2013; Schomakers and Ziefle, 2019). This makes utility-privacy considerations of particular interest for future research. Furthermore, comparing data sharing and disclosing appropriateness between human and robot mediators is interesting. The social assistant robot as described in the vignettes has no human equivalent. Visiting caregivers or a watchful neighbor might be closest. Although, our robot mediator has no human equivalent, it might be interesting to learn more about how the embodiment (e.g., compare to a human) influences the privacy preferences of people.

With our study, we have taken a step towards understanding people’s privacy expectations for robot mediators and determining how to design them to achieve privacy-appropriate operation.

## Data Availability

The raw data supporting the conclusions of this article will be made available by the authors, without undue reservation.
