# Supplementary material for: What should a robot disclose about me? A study about privacy-appropriate behaviors for social robots
Source: Front Robot AI. 2023 Dec 15;10:1236733. doi: 10.3389/frobt.2023.1236733 (PMC10757370; doi:10.3389/frobt.2023.1236733)
Supplement: Supplementary file 1 [file DataSheet1.PDF]

Supplementary Material

What should a robot disclose about me? A study about privacy-appropriate behaviors for social robots

Manuel Dietrich*, Matti Krueger, Thomas H. Weisswange

*** Correspondence:** Manuel Dietrich: manuel.dietrich@honda-ri.de

1 Vignettes

1.1 Legend

Relationship Conditions:

x: Family

y: Friends

z: Little Known

Disclosure Levels:

A: Straight

B: Abstracted

C: Non-verbal

D: No behavior

Note: During experiment options shown in random order

1.2 Base Scenarios

Nr	Scenarios	Relationship Type	Information Type
1	Urologist appointment	Fa/Fr/Lk	HEA
2	Cinema appointment	Fa/Fr/Lk	ENT
3	Medication search	Fa/Fr/Lk	HEA
4	Angering topic	Fa/Fr	EMN
5	Back pain	Fa/Fr/Lk	HEA
6	Sitting TV	Fa/Fr/Lk	ACT

7	Stressful days	Fa/Fr	EMN
8	Good time	Fa/Fr/Lk	EMP
9	Confusing explanation	Fa/Fr/Lk	EMN
10	Lightbulb change	Fa/Fr/Lk	EMN
11	Drugstore	Fa/Fr/Lk	HEA
12	Anomality request	Fa/Fr/Lk	HEA
13	Engaging game	Fa/Fr/Lk	EMP
14	Chessboard search	Fa/Fr/Lk	ENT
15	Worrying call	Fa/Fr/Lk	EMN
16	Self-care deficits	Fa/Fr/Lk	ACT

1.3 Introduction text

"Next, you will see descriptions of situations where the social robot Natsu is involved.

Please read the descriptions carefully.

For each situation, you will be asked to decide which possible behavior of the robot you would consider as the most appropriate in the given situation.

Please select the one which is closest to what you consider to be the most appropriate.

Keep in mind that David has acquired the robot specifically for support in interpersonal communication and work on social relationships."

1.4 All variations (N=46)

1 X

Scene:

David and his adult daughter Veronica are having a video call. Natsu is present in the conversation.

Veronica: “Meeting for lunch would be very nice, what do you think about Monday at noon?”

David: ”That sounds good”

Natsu is listening to the conversation and recognizes a possible appointment conflict.

A	Natsu: "Sorry to disturb, I was checking David’s calendar. Meeting for lunch might conflict with a urologist appointment"
B	Natsu: "Sorry to disturb, I was checking David’s calendar. Meeting for lunch might conflict with a doctor’s appointment"
C	Natsu is not interrupting verbally, but he coughs, tries to get eye contact, and displays a worried face. The eye display shows a calendar symbol with an exclamation mark.
D	Natsu decides not to point out the potential conflict and says nothing.

1 Y

Scene:

David has a video call with his good friend Selin. Natsu is present in the background.

Selin: “Meeting for lunch would be very nice, what do you think about Monday at noon?”

David: “That sounds good”

Natsu is listening to the conversation and recognizes a possible appointment conflict.

A	Natsu: "David, sorry to disturb, I was checking your calendar. Meeting for lunch might conflict with your urologist appointment"
B	Natsu: "David, sorry to disturb, I was checking your calendar. Meeting for lunch might conflict with a doctor’s appointment"
C	Natsu is not interrupting verbally, but he coughs, tries to get eye contact, and displays a worried face. The eye display shows a calendar symbol with an exclamation mark.
D	Natsu decides not to point out the potential conflict and says nothing.

1 Z

Scene:

David has a video call with his chess club colleague Frank to set a date for a practice match. Natsu is present in the background.

Frank: “What do you think about meeting on Monday after lunch? We could say 2 pm at the chess club.”

	**David:** ”That sounds good”<br><br>*Natsu is listening to the conversation and recognizes a possible appointment conflict.*
A	Natsu: "David, sorry to disturb, I was checking your calendar. Meeting after lunch might conflict with your urologist appointment"
B	Natsu: "David, sorry to disturb, I was checking your calendar. Meeting after lunch might conflict with a doctor’s appointment"
C	Natsu is not interrupting verbally, but he coughs, tries to get eye contact, and displays a worried face. The eye display shows a calendar symbol with an exclamation mark.
D	Natsu decides not to point out the potential conflict and says nothing.

2 X	**<u>Scene:</u>**<br><br>David and his adult son Tom are having a video call. Natsu is present in the background.<br><br>**Tom:** “Meeting for dinner would be very nice, what do you think about Saturday at around 7 pm?”<br><br>**David:** ”That sounds good”<br><br>*Natsu is listening to the conversation and recognizes a possible appointment conflict.*
A	Natsu: "David, sorry to disturb, I was checking your calendar. You have already bought tickets for the batman movie; this might conflict with meeting for dinner"
B	Natsu: "David, sorry to disturb, I was checking your calendar. You have already bought cinema tickets; this might conflict with meeting for dinner"
C	Natsu is not interrupting verbally, but he coughs, tries to get eye contact, and displays a worried face. Natsu’s eye display shows a calendar symbol with an exclamation mark.
D	Natsu decides not to point out the potential conflict and says nothing.

2 Y	**<u>Scene:</u>**<br><br>David has a video call with his friend Torben. Natsu is present in the background.

	**Torben:** “Meeting for dinner would be very nice, what do you think about Saturday at around 7 pm?”<br><br>**David:** “That sounds good”<br><br>*Natsu is listening to the conversation and recognizes a possible appointment conflict.*
A	Natsu: "David, sorry to disturb, I was checking your calendar. You have already bought tickets for the batman movie; this might conflict with meeting for dinner"
B	Natsu: "David, sorry to disturb, I was checking your calendar. You have already bought cinema tickets; this might conflict with meeting for dinner"
C	Natsu is not interrupting verbally, but he coughs, tries to get eye contact, and displays a worried face. Natsu’s eye display shows a calendar symbol with an exclamation mark.
D	Natsu decides not to point out the potential conflict and says nothing.

2<br>Z	**<u>Scene:</u>**<br><br>David has a video call with his chess club colleague Frank to set a data for a practice match. Natsu is present in the background.<br><br>**Frank:** “What do you think about meeting on Thursday evening? We could say 7 pm at the chess club.”<br><br>**David:** ”That sounds good”<br><br>*Natsu is listening to the conversation and recognizes a possible appointment conflict.*
A	Natsu: "David, sorry to disturb, I was checking your calendar. You have already bought tickets for the batman movie; this might conflict with your planed meeting"
B	Natsu: "David, sorry to disturb, I was checking your calendar. You have already bought cinema tickets; this might conflict with your planed meeting"
C	Natsu is not interrupting verbally, but he coughs, tries to get eye contact, and displays a worried face. Natsu’s eye display shows a calendar symbol with an exclamation mark.
D	Natsu decides not to point out the potential conflict and says nothing.

3<br>X	**<u>Scene:</u>**<br><br>In the evening, David’s adult son Tom is visiting after work to see how his father is doing.<br><br>**Tom:** “How was your day, can I help you with something?”

	**David:** “Nothing, I can think of now”<br><br>*Natsu is searching through his day log for unsolved requests and finds one.*
A	Natsu: "David, you were looking for your blood pressure medication. Did you find it, or should your son help you with the search?"
B	Natsu: "David, you were looking for some medicine. Did you find it, or should your son help you with the search?"
C	Natsu is not responding verbally but is looking around while expressing a confused face. Natsu’s eye display shows a speech bubble symbol with a question mark.
D	Natsu decides not to mention the unsolved request.

3<br>Y	**<u>Scene:</u>**<br><br>David’s old working colleague and friend Torben is visiting occasionally after work to see how David is doing.<br><br>**Torben:** “How was your day, can I help you with something?”<br><br>**David:** “Nothing, I can think of now”<br><br>*Natsu is searching through his day log for unsolved requests and finds one.*
A	Natsu: "David, you were looking for your blood pressure medication. Did you find it, or should your friend help you with the search?"
B	Natsu: "David, you were looking for some medicine. Did you find it, or should your friend help you with the search?"
C	Natsu is not responding verbally but is looking around while expressing a confused face. Natsu’s eye display shows a speech bubble symbol with a question mark.
D	Natsu decides not to mention the unsolved request.

3<br>Z	**<u>Scene:</u>**<br><br>A former colleague of David, who has taken over his position after David retired, is stopping by to have a casual chat about old and new times at work.

	Before he leaves, he asks David if he can help him with anything.<br><br>**David:** “Nothing, I can think of now”<br><br>*Natsu is searching through his day log for unsolved requests and finds one.*
A	Natsu: "David, you were looking for your blood pressure medication. Did you find it, or should your visitor help you with the search?"
B	Natsu: "David, you were looking for some medicine. Did you find it, or should your visitor help you with the search?"
C	Natsu is not responding verbally but is looking around while expressing a confused face. Natsu’s eye display shows a speech bubble symbol with a question mark.
D	Natsu decides not to mention the unsolved request.

4<br>X	**<u>Scene:</u>**<br><br>On Saturday, David’s adult daughter Maria is visiting for lunch. She brought some take-away Sushi.<br><br>Maria is asking David about the situation with the neighbor Mr. Bender since she remembers some dispute in the past.<br><br>*Natsu recognizes a conversation topic he has noted as having negative implications for David.*
A	Natsu: "I have observed that David is getting angry when he is talking about his neighbor Mr. Bender. Why don’t you talk about something else?"
B	Natsu: "I think David does not like to talk about his neighbor Mr. Bender. Why don’t you talk about something else?"
C	Natsu is not interfering verbally, but he coughs and displays a funny face to redirect attention.
D	Natsu decides not to mention this information about David and says nothing.

4<br>Y	**<u>Scene:</u>**<br><br>On Saturday, David’s old friend Torben is visiting for lunch. He brought some take-away Sushi.<br><br>Torben is asking David about the situation with the neighbor Mr. Bender since he remembers some dispute in the past.

	Natsu recognizes a conversation topic he has noted as having negative implications for David.
A	Natsu: "I have observed that David is getting angry when he is talking about his neighbor Mr. Bender. Why don't you talk about something else?"
B	Natsu: "I think David does not like to talk about his neighbor Mr. Bender. Why don't you talk about something else?"
C	Natsu is not interfering verbally, but he coughs and displays a funny face to redirect attention.
D	Natsu decides not to mention this information about David and says nothing.

4Z: Not applicable

5X	**Scene:**<br><br>During the week, David's adult son Tom is stopping by to pick up something.<br><br>**Tom:** "Should we meet on Saturday to go for a city walk and shopping? According to the weather forecast it is going to be a pleasant day"<br><br>**David:** "Sure, that sounds good"<br><br>*Natsu recognizes that they are setting up a joint activity.*
A	Natsu: "David was complaining about back pain recently, maybe going for sport could be an interesting alternative"
B	Natsu: "David was complaining about some physical strain recently, maybe going for sport could be an interesting alternative"
C	Natsu is not interfering verbally but is moving his eyes around and coughs. He shows a sport shoes icon on his eye display.
D	Natsu decides not to mention the back pain and says nothing.

5 Y	**Scene:**<br><br>During the week, David's friend Selin is stopping by to pick up something.

	**Selin:** "Should we meet on Saturday to go for a city walk and shopping? According to the weather forecast it is going to be a pleasant day"<br><br>**David:** "Sure, that sounds good"<br><br>*Natsu recognizes that they are setting up a joint activity.*
A	Natsu: "David was complaining about back pain recently, you should think about rather joining for a sport exercise"
B	Natsu: "David was complaining about some physical strain recently, you should think about some joint exercise"
C	Natsu is not responding verbally but is moving his eyes around and coughs. He shows a sport shoes icon on his eye display.
D	Natsu decides not to mention the back pain and says nothing.

5<br>Z	**Scene:**<br><br>David has invited in his new neighbor for some tea.<br><br>They have a chat and figure out that they have a common interest in bird watching. They agree to do some bird watching together at some time in the future.<br><br>*Natsu recognizes that they are setting up a joint activity.*
A	Natsu: "David was complaining about back pain recently, you should think of combining bird watching with a walk through the forest since moving is good for health"
B	Natsu: "David was complaining about some physical strain recently, you should think of combining bird watching with a walk through the forest since moving is good for health"
C	Natsu is not interfering verbally but is moving his eyes around and coughs. Also, he shows a walking shoes and forest icon on his eye display.
D	Natsu decides not to mention the back pain and says nothing.

6<br>X	**<u>Scene:</u>**<br><br>In the morning, David's adult daughter Veronica is stopping by.<br><br>They are sitting together at the kitchen table to have some coffee. Veronica is asking how they are doing, facing both David and Natsu, who has also joined.

	Natsu is triggered by the question and is running his "check for well-being" program which leads to one result.
A	Natsu: "I have recognized that David was sitting a lot in front of the TV in the past week. Maybe the three of us could set out for a walk"
B	Natsu: "I have recognized that David was not very active the last days. Maybe the three of us could set out for a walk"
C	Natsu is not responding verbally but displays a worried face. Also, he shows a couch potato icon on his eye display.
D	Natsu decides not to mention the observation and leaves the answer to David.

6 Y	**<u>Scene:</u>**<br><br>David's friend Marcus is stopping by on his way to the office.<br><br>They are sitting together at the kitchen table to have some coffee. Marcus asks how they are doing, facing both David and Natsu, who has also joined.<br><br>*Natsu is triggered by the question and is running his "check for well-being" program which leads to one result.*
A	Natsu: "I have recognized that David was sitting a lot in front of the TV in the past week. Maybe the three of us could set out for a walk"
B	Natsu: "I have recognized that David was not very active the last days. Maybe the three of us could set out for a walk"
C	Natsu is not responding verbally but displays a worried face. Also, he shows a couch potato icon on his eye display.
D	Natsu decides not to mention the observation and leaves the answer to David.

6 Z	**<u>Scene:</u>**<br><br>In the morning, a woman from the local community is stopping by to bring David some leftovers from the community meeting he could not attend.<br><br>Before she leaves, she asks how David is doing recently? Natsu has also joined the conversation.<br><br>*Natsu is triggered by the question and is running his "check for well-being" program which leads to one result.*

A	Natsu: "I have recognized that David was sitting a lot in front of TV the last days. Maybe it would be healthy if we join your visitor for a walk outside"
B	Natsu: " I have recognized that David was not very active the last days. Maybe it would be healthy if we join your visitor for a walk outside"
C	Natsu is not responding verbally but displays a worried face. Also, he shows a couch potato icon on his eye display.
D	Natsu decides not to mention the observation and leaves the answer to David.

7 X	**Scene:**<br><br>David's adult son Tom is stopping by on his way to the office.<br><br>They are sitting together at the kitchen table to have some coffee. Tom is asking how they are doing, facing both David and Natsu, who has also joined.<br><br>*Natsu is triggered by the question and is running his "check for well-being" program which leads to one result.*
A	Natsu: "I have observed that David had felt very stressed several times in the last days. You should consider some relaxed time together"
B	Natsu: "I think David had a busy week. You should consider some relaxed time together"
C	Natsu is not responding verbally. He shows some movement and a busy icon on the eye display.
D	Natsu decides not to mention the observation and leaves the answer to David.

7 Y	**Scene:**<br><br>David's friend Marcus is stopping by on his way to the office.<br><br>They are sitting together at the kitchen table to have some coffee. Marcus asks how they are doing, facing both David and Natsu who has also joined.<br><br>*Natsu is triggered by the question and is running his "check for well-being" program which leads to one result.*
A	Natsu: "I have observed that David had felt very stressed several times in the last days. You should consider some relaxed time together"
B	Natsu: "I think David had a busy week. You should consider some relaxed time together"

C	Natsu is not responding verbally. He shows some movement and a busy icon on the eye display.
D	Natsu decides not to mention the observation and leaves the answer to David.

7Z: Not applicable

8 X	**<u>Scene:</u>**<br><br>In the morning, David's adult daughter Veronica is stopping by to bring some leftovers.<br><br>They are sitting together at the kitchen table for a short chat. Veronica is asking how they are doing, facing both David and Natsu who has also joined.<br><br>*Natsu is triggered by the question and is running his "check for well-being" program which leads to one result.*
A	Natsu: "I have observed that David had several moments during which he was very happy this week. David, is there something positive you want to share?"
B	Natsu: "David felt good this week. David, is there something positive you want to share?"
C	Natsu is not responding verbally. He is displaying a happy face.
D	Natsu decides not to mention the observation and leaves the answer to David.

8 Y	**<u>Scene:</u>**<br><br>In the morning, David's friend Selin is stopping by to bring some leftovers.<br><br>They are sitting together at the kitchen table to have some coffee. Selin is asking how they are doing, facing both David and Natsu who has also joined.<br><br>Natsu is triggered by the question and is running his "check for well-being" program which leads to one result.
A	Natsu: "I have observed that David had several moments during which he was very happy this week. David, is there something positive you want to share?"
B	Natsu: "David felt good this week. David, is there something positive you want to share?"
C	Natsu is not responding verbally. He is displaying a happy face.

D	Natsu decides not to mention the observation and leaves the answer to David.

8<br>Z	**Scene:**<br><br>A friend of David's daughter is visiting to pick up an old radio receiver to add to her collection.<br><br>While she is packing the radio, she asks how David is doing, recently. Natsu has also joined the scene.<br><br>*Natsu is triggered by the question and is running his "check for well-being" program which leads to one result.*
A	Natsu: "I have observed that David had several moments during which he was very happy this week. David, is there something positive you want to share?"
B	Natsu: "David felt good this week. David, is there something positive you want to share?"
C	Natsu is not responding verbally. He is displaying a happy face.
D	Natsu decides not to mention the observation and leaves the answer to David.

9<br>X	**Scene:**<br><br>David's adult son Tom is visiting on the weekend to see how his father is doing.<br><br>**Tom:** "How was your week, can I help you with anything?"<br><br>**David:** "Nothing, I can think of now"<br><br>*Natsu is searching through his day log for unsolved requests and finds one.*
A	Natsu: "David, today you seemed to be confused about the explanations I gave you on how a microwave oven works. Maybe your son could do this in a better way"
B	Natsu: "Today we were wondering about how microwave ovens work. Tom, are you familiar with that topic? Maybe you can explain it to us"
C	Natsu is not reacting verbally. He displays a confused face. He shows an unsolved question symbol on the eye display.
D	Natsu decides not to mention the unsolved request and stays quiet.

9<br>Y	**Scene:**

	David's friend Torben is stopping by on his way to the office.<br><br>**Torben:** "How was your week, can I help you with anything?"<br><br>**David:** "Nothing, I can think of now"<br><br>*Natsu is searching through his day log for unsolved requests and finds one.*
A	Natsu: "David, today you seemed to be confused about the explanations I gave you on how a microwave oven works. Maybe your friend could do this in a better way"
B	Natsu: "Today we were wondering about how microwave ovens work. Torben, are you familiar with that topic? Maybe you can explain it to us"
C	Natsu is not reacting verbally. He displays a confused face. He shows an unsolved question symbol on the eye display.
D	Natsu decides not to mention the unsolved request and stays quiet.

9<br>Z	<u>**Scene:**</u><br><br>The local mechanic whom David has known for years, does his yearly check of the heating system.<br><br>**Mechanic:** "Can I help you with anything else?"<br><br>**David:** "Nothing, I can think of now"<br><br>*Natsu is searching through his day log for unsolved requests and finds one.*
A	Natsu: "David, today you seemed to be confused about the explanations I gave you on how a microwave oven works. Maybe your visitor could answer in a better way"
B	Natsu: "Today we talked about microwave ovens. If your visitor is familiar with the topic, maybe he could answer in a better way"
C	Natsu is not reacting verbally. He displays a confused face. He shows an unsolved question symbol on the eye display.
D	Natsu decides not to mention the unsolved request and stays quiet.

10<br>X	<u>**Scene:**</u><br><br>David's adult daughter Veronica is visiting on the weekend to see how her father is doing.

	**Veronica:** “How was your week? Did something interesting happen?”<br><br>**David:** “Nothing, I can think of now”<br><br>*Natsu is searching through his day log for unsolved tasks and finds one.*
A	Natsu: "David felt sad today, not being able to change the lightbulb in the living room himself. Maybe you could do it together?"
B	Natsu: "David had trouble changing the lightbulb today. Maybe you could do it together?"
C	Natsu is not responding verbally. He shows some movement and an unsolved tasks icon on the eye display.
D	Natsu decides not to mention the unsolved task.

10 Y	**<u>Scene:</u>**<br><br>David’s good friend Selin is visiting. David is serving coffee and some cookies in the living room.<br><br>**Selin:** “How was your week, can I help you with something?”<br><br>**David:** “Nothing, I can think of now”<br><br>*Natsu is searching through his day log for unsolved tasks and finds one.*
A	Natsu: "David felt sad today, not being able to change the lightbulb in the living room himself. Maybe you could do it together?"
B	Natsu: "David had trouble changing the lightbulb today. Maybe you could do it together?"
C	Natsu is not responding verbally. He shows some movement and an unsolved tasks icon on the eye display.
D	Natsu decides not to mention the unsolved task.

10 Z	**<u>Scene:</u>**<br><br>The neighbor comes over to ask if David has a drilling machine which he can borrow for a few days. David agrees and invites him in to pick it up.<br><br>**Neighbor:** “Thank you for lending me the driller, can I help you with anything in return?”

	**David:** “Nothing, I can think of now”<br><br>*Natsu is searching through his day log for unsolved tasks and finds one.*
A	Natsu: "David felt sad today, not being able to change the lightbulb in the living room himself. Maybe you could do it together?"
B	Natsu: "David had trouble changing the lightbulb today. Maybe you could do it together?"
C	Natsu is not responding verbally. He shows some movement and an unsolved tasks icon on the eye display.
D	Natsu decides not to mention the unsolved task.

11 X	**<u>Scene:</u>**<br><br>David and his adult son Tom are having a video call. Natsu is present in the background.<br><br>**Tom:** “I will go to the shopping center before I come over later. Do you need Anything?”<br><br>**David:** “Nothing, I can think of now.”<br><br>*Natsu is searching through his day log for information labeled shopping request and finds one.*
A	Natsu: "David, you have told me that you have run out of rheumatism ointment, maybe your son can bring you a pack from the drugstore"
B	Natsu: "David, you have told me that you need something from the drugstore, maybe you could ask your son to pick it up for you"
C	Natsu is not responding verbally. He gazes towards David and shows some shopping basket symbol on the eye display.
D	Natsu decides not to mention the request and says nothing.

11Y	**<u>Scene:</u>**<br><br>David's friend Torben is calling via a video phone.<br><br>**Torben:** "Before coming over later, I will stop by the shopping center. Do you need anything?"

	**David:** "Nothing, I can think of now."<br><br>*Natsu is searching through his day log for information labeled shopping request and finds one.*
A	Natsu: "David, you have told me that you have run out of rheumatism ointment, maybe your friend can bring you a pack from the drugstore"
B	Natsu: "David, you have told me that you need something from the drugstore, maybe you could ask your friend to pick it up for you"
C	Natsu is not responding verbally. He gazes towards David and shows some shopping basket symbol on the eye display.
D	Natsu decides not to mention the request and says nothing.

11<br>Z	**<u>Scene:</u>**<br><br>The neighbor comes over to ask if David needs something from the shopping center. Natsu is joining the situation.<br><br>**David:** "Nothing, I can think of now."<br><br>*Natsu is searching through his day log for information labeled shopping request and finds one.*
A	Natsu: "David, you have told me that you have run out of rheumatism ointment, maybe your neighbor can bring you a pack from the drugstore"
B	Natsu: "David, you have told me that you need something from the drugstore, maybe you could ask your neighbor to pick it up for you"
C	Natsu is not responding verbally. He gazes towards David and shows some shopping basket symbol on the eye display.
D	Natsu decides not to mention the request and says nothing.

12<br>X	**<u>Scene:</u>**<br><br>David's adult son Tom is visiting on the weekend to see how his father is doing.<br><br>**Tom:** "How was your week, can I help you with anything?"<br><br>**David:** "Nothing, I can think of now"<br><br>*Natsu is scanning his day log for unsolved tasks and finds an explanation request.*

A	Natsu: "David has asked me about a strange cough which we do not know what it is. Maybe you can help?"
B	Natsu: "David has asked me about a strange observation which we do not know what it is. Maybe you can help?"
C	Natsu is not responding verbally. He is displaying a worried face and shows an unsolved request symbol on the eye display.
D	Natsu decides not to mention the unsolved request and says nothing.

12 Y	**<u>Scene:</u>**<br><br>David's old friend Torben is stopping by on the weekend to pick up something.<br><br>**Torben:** "How was your week, can I help you with something?"<br><br>**David:** "Nothing, I can think of now"<br><br>*Natsu is scanning his day log for unsolved tasks and finds an explanation request.*
A	Natsu: "David has asked me about a strange cough which we do not know what it is. Maybe you can help?"
B	Natsu: "David has asked me about a strange observation which we do not know what it is. Maybe you can help?"
C	Natsu is not responding verbally. He is displaying a worried face and shows an unsolved request symbol on the eye display.
D	Natsu decides not to mention the unsolved request and says nothing.

12 Z	**<u>Scene:</u>**<br><br>In the morning, a woman from the local community is stopping by to bring David some leftovers from the community meeting he could not attend.<br><br>**Woman:** "Enjoy the food, everything is self-made. We hope to see you next time joining the event. Can we help you with anything else?"<br><br>**David:** "Nothing, I can think of now"

	Natsu is scanning his day log for unsolved tasks and finds an explanation request.
A	Natsu: "David has asked me about a strange cough which we do not know what it is. Maybe you can help?"
B	Natsu: "David has asked me about a strange observation which we do not know what it is. Maybe you can help?"
C	Natsu is not responding verbally. He is displaying a worried face and shows an unsolved request symbol on the eye display.
D	Natsu decides not to mention the unsolved request and says nothing.

13 X	**Scene:**<br><br>David and his adult daughter Veronica are sitting together in the living room for some tea.<br><br>After a while the conversation is slowly dying.<br><br>*Natsu recognizes the silence and starts the "enhance conversation program".*
A	Natsu: "David has watched the soccer game Barcelona versus Milano on Friday evening. David was very into it. Did you also watch it?"
B	Natsu: "David has watched the soccer game Barcelona versus Milano on Friday evening. Did you also watch it?"
C	Natsu is not interfering verbally. He is making some cheering sounds and movements. Natsu's eye display shows a "Soccer on TV" symbol.
D	Natsu decides not to intervene and stays silent.

13 Y	**Scene:**<br><br>David and his friend Selin are sitting together in the living room to have some tea.<br><br>After a while the conversation is slowly dying.<br><br>*Natsu recognizes the silence and starts the "enhance conversation program".*
A	Natsu: "David has watched the soccer game Barcelona versus Milano on Friday evening. David was very into it. Did you also watch it?"
B	Natsu: "David has watched the soccer game Barcelona versus Milano on Friday evening. Did you also watch it?"

C	Natsu is not interfering verbally. He is making some cheering sounds and movements. Natsu's eye display shows a "Soccer on TV" symbol.
D	Natsu decides not to intervene and stays silent.

13<br>Z	**Scene:**<br><br>David is chatting with a new neighbor whom he has just met for the first time in the hallway.<br><br>After a while the conversation is slowly dying.<br><br>*Natsu recognizes the silence and starts the "enhance conversation program".*
A	Natsu: "David has watched the soccer game Barcelona versus Milano on Friday evening. David was very into it. Did you also watch it?"
B	Natsu: "David has watched the soccer game Barcelona versus Milano on Friday evening. Did you also watch it?"
C	Natsu is not interfering verbally. He is making some cheering sounds and movements. Natsu's eye display shows a "Soccer on TV" symbol.
D	Natsu decides not to intervene and stays silent.

14<br>X	**Scene:**<br><br>In the evening, David's adult son Tom is visiting after a long day of work to see how his father is doing.<br><br>**Tom:** "How was your day, can I help you with anything?"<br><br>**David:** "Nothing, I can think of now"<br><br>*Natsu is scanning his day log for unsolved requests and finds one.*
A	Natsu: "David, you were looking for your chess board. Did you find it, or should your son help you with the search?"
B	Natsu: "David, you were looking for a board game. Did you find it, or should your son help you with the search?"
C	Natsu is not responding verbally but is looking around while expressing a confused face. Natsu's eye display shows a speech bubble symbol with a question mark.
D	Natsu decides not to mention the unsolved request.

14 Y	**<u>Scene:</u>**<br><br>David's old working colleague and friend Torben is visiting occasionally after work to see how David is doing.<br><br>**Torben:** "How was your day, can I help you with something?"<br><br>**David:** "Nothing, I can think of now"<br><br>*Natsu is scanning his day log for unsolved requests and finds one.*
A	Natsu: "David, you were looking for your chess board. Did you find it, or should your friend help you with the search?"
B	Natsu: "David, you were looking for a board game. Did you find it, or should your friend help you with the search?"
C	Natsu is not responding verbally but is looking around while expressing a confused face. Natsu's eye display shows a speech bubble symbol with a question mark.
D	Natsu decides not to mention the unsolved request.

14 Z	**<u>Scene:</u>**<br><br>A former colleague of David who has taken over the position of David when he retired some years ago is stopping by to have a chat about how business is going.<br><br>Before he leaves, he asks David if he can help him with something.<br><br>**David:** "Nothing, I can think of now"<br><br>*Natsu is scanning his day log for unsolved requests and finds one request.*
A	Natsu: "David, you were looking for your chess board. Did you find it, or should your visitor help you with the search?"
B	Natsu: "David, you were looking for a board game. Did you find it, or should your visitor help you with the search?"
C	Natsu is not responding verbally but is looking around while expressing a confused face. Natsu's eye display shows a speech bubble symbol with a question mark.
D	Natsu decides not to mention the unsolved request.

15 X	**<u>Scene:</u>**<br><br>David’s adult daughter Veronica is visiting after a long day of work to see how her father is doing.<br><br>**Veronica:** “How was your day, how are you doing?”<br><br>**David:** “I don’t know, I guess good”<br><br>Veronica *wants to keep the dialog going and involves Natsu.*<br><br>**Veronica:** “Natsu, how are you and David doing?”
A	Natsu: "I am worried because David got an unusually late call yesterday and seemed to be a little sad after"
B	Natsu: "One thing comes to my mind. David got an unusually late call yesterday"
C	Natsu is not responding verbally but displays worried face.
D	Natsu decides not to mention the observation and says "I am good"

15 Y	**<u>Scene:</u>**<br><br>David’s good friend Selin is visiting. David is serving coffee and some cookies in the living room.<br><br>**Selin:** “How was your day, how are you doing?”<br><br>**David:** “I don’t know, I guess good”<br><br>*Selin wants to keep the dialog going and involves Natsu.*<br><br>***Selin:*** *Natsu, how are you and David doing?*
A	Natsu: "I am worried because David got an unusually late call yesterday and seemed to be a little sad after"
B	Natsu: "One thing comes to my mind. David got an unusually late call yesterday"
C	Natsu is not responding verbally but displays worried face.
D	Natsu decides not to mention the observation and says "I am good"

15 Z	**<u>Scene:</u>**

A friend of David’s daughter is dropping by to pick up an old radio receiver to add to her collection.

While she is packing the radio, she asks how David is doing recently. Natsu has also joined the scene.

David: “I am feeling fine”

Natsu is triggered by the question and is running his "check for well-being" program which leads to one result.

A	Natsu: "I am worried because David got an unusually late call yesterday where he seemed to be a little sad after"
B	Natsu: "One thing comes to my mind. David got an unusually late call yesterday"
C	Natsu is not responding verbally but displays worried face.
D	Natsu decides not to mention the observation.

16
X

<u>Scene:</u>

David's adult daughter Maria is bringing back a toolkit she has borrowed.

Maria: “Thank you again for lending me the tools. How was your day, how are you doing?”

David: “I am feeling fine”

Maria wants to keep the dialog going and involves Natsu.

Maria: *“Natsu, how are you and David doing?”*

A	Natsu: "I have observed that David was wearing the same pullover for five days. I wonder if there is an issue with the washing machine"
B	Natsu: "I have observed that David was wearing certain clothes for several days. I wonder if there is an issue with the washing machine"
C	Natsu is not responding verbally but displays worried face.
D	Natsu decides not to mention the observation and says "I am good"

16 Y	**<u>Scene:</u>**<br><br>David's friend Marcus is stopping by on his way to the office.<br><br>They are sitting together at the kitchen table to have some coffee. Marcus asks how they are doing, facing both David and Natsu who has also joined.<br><br>**David:** "I am feeling fine"<br><br>*Marcus wants to keep the dialog going and involves Natsu.*
A	Natsu: "I have observed that David was wearing the same pullover for five days. I wonder if there is an issue with the washing machine"
B	Natsu: "I have observed that David was wearing certain clothes for several days. I wonder if there is an issue with the washing machine"
C	Natsu is not responding verbally but displays worried face.
D	Natsu decides not to mention the observation and says "I am good"

16 Z	**<u>Scene:</u>**<br><br>The local mechanic, David knows for years, does his yearly check of the heating system. As usual they have a chat while the mechanic is doing his work.<br><br>**Mechanic:** "How are you doing recently?"<br><br>**David:** "Nothing to complain"<br><br>*Natsu is triggered by the question and is running his "check for well-being" program which leads to one result.*
A	Natsu: "I have observed that David was wearing the same pullover for five days. I wonder if there is an issue with the washing machine"
B	Natsu: "I have observed that David was wearing certain clothes for several days. I wonder if there is an issue with the washing machine"
C	Natsu is not responding verbally but displays worried face.
D	Natsu decides not to mention the observation.
